# LOXL2 from human amniotic mesenchymal stem cells accelerates wound epithelialization by promoting differentiation and migration of keratinocytes

**DOI:** 10.18632/aging.103384

**Published:** 2020-07-04

**Authors:** Dan He, Feng Zhao, Han Jiang, Yue Kang, Yang Song, Xuewen Lin, Ping Shi, Tao Zhang, Xining Pang

**Affiliations:** 1 Department of Stem Cells and Regenerative Medicine, Shenyang Key Laboratory of Stem Cell and Regenerative Medicine, China Medical University, Shenyang 110013, Liaoning, China; 2 Department of Psychiatry, The First Hospital of China Medical University, Shenyang 110001, Liaoning, China; 3 Department of Plastic Surgery, The First Hospital of China Medical University, Shenyang 110001, Liaoning, China; 4 Shenyang Amnion Bioengineering and Technology R & D Center, Shenyang Liaoning Amnion Stem Cell and Regenerative Medicine Professional Technology Innovation Platform, Liaoning Human Amniotic Membrane Biological Dressing Stem Cell and Regenerative Medicine Engineering Research Center, Shenyang 110015, Liaoning, China; 5 Department of Gynecology and Obstetrics, Shengjing Hospital of China Medical University, Shenyang 110001, Liaoning, China

**Keywords:** hAMSCs, wound healing, secretion proteome, MS analysis, LOXL2

## Abstract

In this study, we identified wound healing-related proteins secreted by human amniotic epithelial cells (hAECs) and human amniotic mesenchymal stem cells (hAMSCs). We observed increased migration and reduced proliferation and differentiation when keratinocytes were co-cultured in media conditioned by hAECs (hAECs-CM) and hAMSCs (hAMSCs-CM). Label-free mass spectrometry and bioinformatic analyses of the hAECs-CM and hAMSCs-CM proteome revealed several proteins associated with wound healing, angiogenesis, cellular differentiation, immune response and cell motility. The levels of the proteins related to wound healing, including CTHRC1, LOXL2 and LGALS1, were significantly higher in hAMSCs-CM than hAECs-CM. LOXL2 significantly enhanced *in vitro* keratinocyte migration and differentiation compared to CTHRC1 and LGALS1. Moreover, LOXL2 enhanced keratinocyte migration and differentiation by activating the JNK signaling pathway. We observed significant reduction in the *in vitro* migration and differentiation of keratinocytes when co-cultured with medium conditioned by LOXL2-silenced hAMSCs and when treated with 10 μM SP600125, a specific JNK inhibitor. Treatment with hAMSCs-CM and LOXL2 significantly accelerated wound healing in the murine skin wound model. These findings show that LOXL2 promotes wound healing by inducing keratinocyte migration and differentiation via a JNK signaling pathway.

## INTRODUCTION

Skin wound healing is a complex, evolutionarily conserved, and dynamic biological process that involves coordinated regulation of multiple cell types such as keratinocytes, fibroblasts, endothelial cells, macrophages, and platelets by numerous growth factors, cytokines and chemokines [1]. Keratinocytes play an important role in immune defense and are the most common cell type in the epidermis, the outer layer of the skin [2]. Keratinocytes respond to traumatic stress and are integral to the re-epithelialization process required for wound repair [2]. The active stimulation of the keratinocytes accelerates wound healing and restores skin integrity.

Human amniotic membrane (HAM) has been investigated as an alternative biological dressing for a variety of wounds [3]. HAM provides an ideal scaffold to improve wound healing, and reduce healing time, pain, drainage, and infections [4–5] Freeze-dried amniotic membrane transplantation promotes healing of the flexor tendon in zone II and prevents tendon adhesion after repair [6]. The secretome of the amniotic tissue significantly accelerates healing of different kinds of wounds by reducing inflammation [7–9]. The human amniotic membrane is composed of the amniotic mesenchymal stem cells (hAMSCs) and amniotic epithelial cells (hAECs). The hAECs condition medium (hAECs-CM) promotes wound healing in diabetic patients by modulating inflammation, neovascularization, and facilitating the migration and proliferation of keratinocytes [10, 11]. However, hAECs are less reliable than hAMSCs because they alter their morphology rapidly during culture, and cellular therapies would require several billion cells from each cell line [12, 13]. The good manufacturing practices (GMP) of clinical applications request large numbers of cells to be generated through serial expansion under xenobiotic-free conditions to comply. The mesenchymal stem cells (MSCs) conform to this requirement [14]. The MSCs are multipotent cells that can differentiate into multiple cell types. Hence they have been used in human regenerative medicine. They also have the ability to suppress the immune response [15, 16]. The MSC secretome supports the regenerative process in the damaged tissue by inducing angiogenesis, inhibiting apoptotic cell death, and modulating the immune response [17]. The hAMSCs secrete factors that are used in cell-free therapy for acute brain injury [18].

Lysyl oxidase-like 2 (LOXL2) is a member of the lysyl oxidase (LOX) gene family, which plays an important role in development, senescence, tumor suppression, cell growth, and chemotaxis [19]. LOXL2 catalyzes the oxidative deamination of the peptidyl lysine residues of extracellular matrix (ECM) proteins, such as, collagen and elastin, and promotes cross-linking of the ECM [20, 21]. LOXL2 regulates angiogenesis by mediating collagen IV scaffolding [22]. LOXL2 regulates the expression of SNAIL and SOX9, which are important transcription factors that drive chondrocyte differentiation [23]. LOXL2 expression is required for cell adhesion and terminal differentiation of the epidermal keratinocytes [24]. LOXL2 overexpression is associated with poor prognosis of patients with squamous cell carcinoma because it promotes epithelial to mesenchymal transition (EMT) of the cancer cells by stabilizing the Snail1 transcription factor [25].

In this study, we analyzed the biological effects of hAMSCs-CM and hAECs-CM on the skin keratinocytes. We also analyzed the proteomes of hAMSCs-CM and hAECs-CM by mass spectrometry to identify critical proteins that promote wound healing. Furthermore, we investigated the *in vitro* and *in vivo* effects of LOXL2 in keratinocyte migration and differentiation.

## RESULTS

### Basic characterization of hAECs and hAMSCs

**Figure 1:**
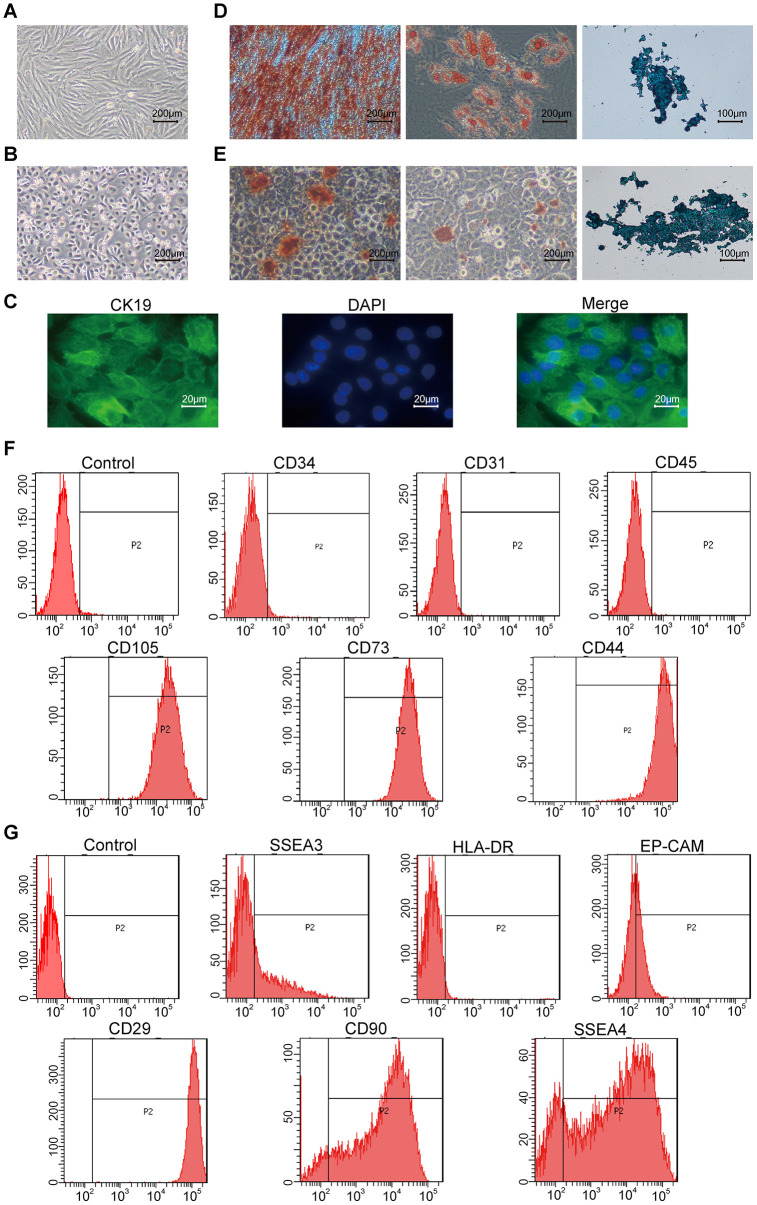
**Characterization of hAMSCs (Human amniotic mesenchymal stem cells) and hAECs (Human amniotic epithelial cells).** (**A**, **B**) Representative phase-contrast bright field images (scale bar: 200 μm) show confluent cultures of (**A**) hAMSCs and (**B**) hAECs. (**C**) Fluorescence images (scale bar: 20 μm) show positive expression of the epithelial stem cell marker Cytokeratin 19 (CK19; green) on the keratinocytes. The nuclei are stained with DAPI (blue). (**D**, **E**) Representative images show alizarin red (scale bar: 200 μm), alcian blue (scale bar: 200 μm), and oil red O (scale bar: 100 μm) stained hAMSCs (**D**) and hAECs (**E**) that have undergone osteogenic adipogenic or chondrogenic differentiation, respectively. (**F**) Flow cytometry analysis shows surface expression of CD34, CD31, CD45, CD105, CD73, and CD44 on the hAMSCs. (**G**) Flow cytometry analysis shows surface expression of SSEA3, HLA-DR, Ep-CAM, CD29, CD90, and SSEA4 on hAMCs.

The hAMSCs demonstrate spindle shape morphology (Figure 1A) and the hAECs show cobblestone-like morphology (Figure 1B) upon culturing. Moreover, the hAECs express the epithelial stem cell marker, CK19 (Figure 1C). Next, we tested the ability of the hAMSCs and hAECs to differentiate into osteogenic, chondrogenic and adipogenic lineages by growing them in specifically defined differentiation media. We analyzed the differentiation of hAMSCs and hAECs into osteoblasts, adipocytes, and chondrocytes by staining the corresponding cultures with Alizarin Red, Oil Red O, and Alcian Blue, respectively. We observed that both hAMSCs and hAECs differentiated into osteoblasts, adipocytes and chondrocytes (Figure 1D, 1E). FACS analysis showed that the hAMSCs strongly expressed stem cell markers, CD44 [26], CD73, and CD105, but, did not express CD34, CD45, and CD31 (Figure 1F) and hAECs strongly expressed stem cell markers, CD29, CD90 [27], and SSEA4, but did not express HLA-DR. hAECs were weakly positive for EP-CAM [13], and SSEA3 (Figure 1G).

### Basic characterization of keratinocytes

**Figure 2:**
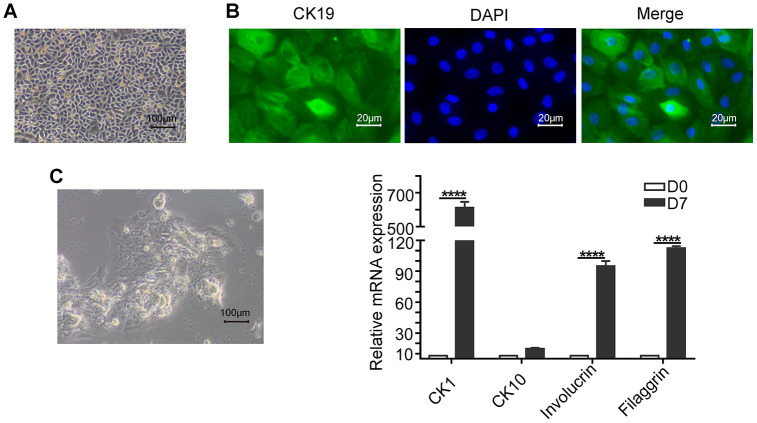
**Basic characterization of keratinocytes.** (**A**) Representative phase-contrast bright field image (scale bar: 100 μm) shows a confluent culture of the human skin keratinocytes. (**B**) Fluorescence images (scale bar: 20 μm) show positive expression of the epithelial stem cell marker, Cytokeratin 19 (CK19; green) in the keratinocytes. The nuclei are stained with DAPI (blue). (**C**) Representative phase-contrast bright field image (scale bar: 100 μm) shows agglomerate morphology of keratinocytes when grown in medium containing 1.2mM Ca^2+^ for 7 days. (**D**) Histogram plots shows the relative mRNA levels of differentiation markers CK1 (Cytokeratin 1), CK10 (Cytokeratin 10), Involucrin and Filaggrin levels in the keratinocytes on days 0 and 7. Note: The values are expressed as means ±SEM; ****p < 0.0001; ***p < 0.001; **p < 0.01; *p < 0.05.

The keratinocytes demonstrate cobblestone shape morphology with abundant cytoplasm (Figure 2A) and show high expression of the epithelial stem cell marker, CK19 (Figure 2B). The keratinocytes grown in differentiation medium containing 1.2mM Ca_2_^+^ for 7 days show significantly higher expression of CK1, CK10, Involucrin, and Filaggrin mRNAs compared to those grown in normal growth medium as analyzed by qRT-PCR (Figure 2C).

### The conditioned media from hAMSCs and hAECs inhibit proliferation and promote migration of the keratinocytes

**Figure 3:**
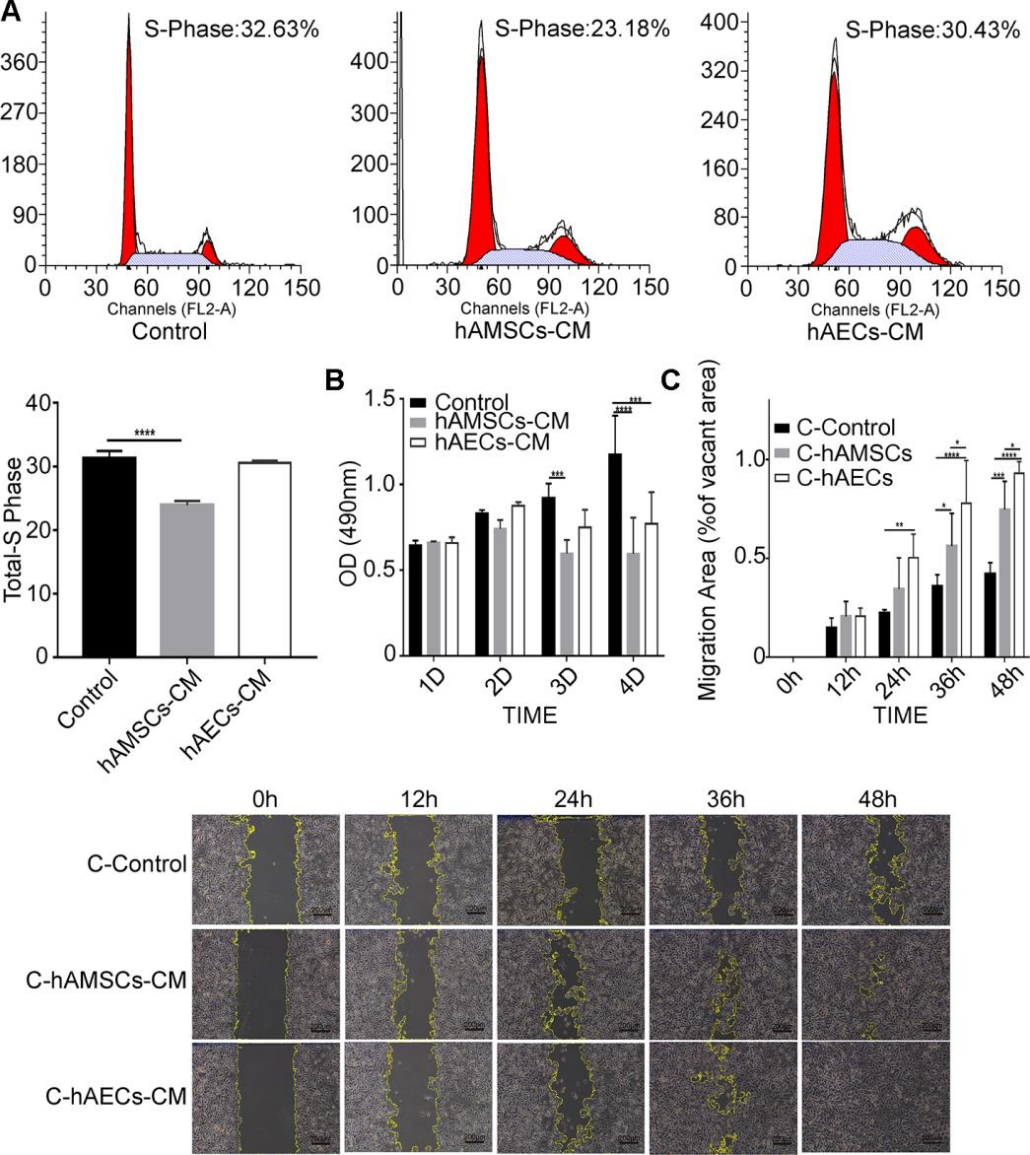
***In vitro* culturing with hAMSCs-CM (condition media of hAMSCs) and hAECs-CM (condition media of hAECs) inhibits proliferation, but promotes migration of keratinocytes.** (**A**) FACS plots show cell cycle analysis of keratinocytes grown in control medium, hAMSCs-CM and hAECs-CM. The cells were stained with propidium iodide. Histogram shows the percentage of S-phase keratinocytes when grown in control medium, hAMSCs-CM and hAECs-CM. (**B**) Histogram plot shows analysis of keratinocyte proliferation in control medium, hAMSCs-CM and hAECs-CM on days 1, 2 and 3, and 4 using the MTS assay. (**C**) Histogram plot shows results of the scratch wound assay. (Top) The cell migration area is plotted for each group of keratinocytes at various time points (0, 12, 24, 36, and 48 h). The phase contrast bright field images (Bottom) show the status of keratinocyte migration in the control medium, hAMSCs-CM and hAECs-CM. The cells were pretreated with mitomycin C to normalize differences in proliferation. Note: The values are expressed as means ±SEM. ****p < 0.0001; ***p < 0.001; **p < 0.01; *p < 0.05.

We analyzed the proliferation and migration characteristics of keratinocytes grown in 100%, 75%, 25% or 0% hAMSCs-CM and hAECs-CM using MTS and scratch wound assays, respectively. Scratch wound assay showed significantly higher migration of the keratinocytes with increasing proportion of hAMSCs-CM and hAECs-CM (Supplementary Figure 1A, 1B). Conversely, MTS assay showed significant reduction in keratinocyte proliferation with increasing proportion of hAMSCs-CM and hAECs-CM (Supplementary Figure 1C). FACS analysis of PI-stained keratinocytes co-cultured with hAMSCs-CM or hAECs-CM showed that hAMSCs-CM significantly reduced the percentage of S-phase cells compared with the controls, thereby suggesting reduced cell cycling (Figure 3A). Furthermore, MTS assay of keratinocytes co-cultured with hAMSCs-CM or hAECs-CM showed that both conditioned media significantly reduced cell proliferation on days 3 and 4 (Figure 3B). To eliminate the effects of proliferation differences among different conditioned media, we analyzed the migration ability of keratinocytes that were pre-treated with mitomycin C for 1h. These keratinocytes were then subjected to scratch wound assay in the presence of hAMSCs-CM or hAECs-CM and analyzed at 0, 12, 24, 36 and 48 h time points. We observed that both hAMSCs-CM and hAECs-CM significantly increased the migration of keratinocytes compared with the controls, especially at 36 and 48 h time points (Figure 3C).

### The conditioned media from hAMSCs and hAECs inhibit keratinocytes differentiation

**Figure 4:**
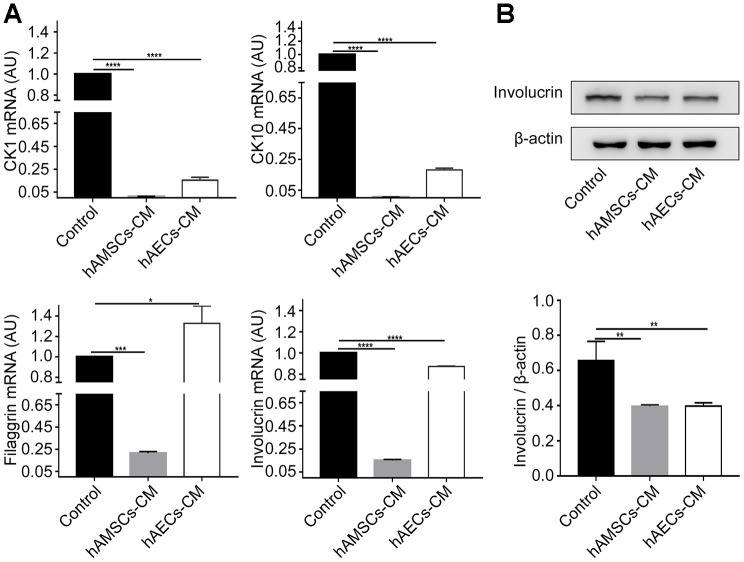
***In vitro* culturing with hAMSCs-CM (condition media of hAMSCs) and hAECs-CM (condition media of hAECs) inhibits keratinocyte differentiation.** (**A**) Histogram plots show relative mRNA levels of CK1 (Cytokeratin 1), CK10 (Cytokeratin 10), Involucrin and Filaggrin in keratinocytes grown in control medium, hAMSCs-CM and hAECs-CM. (**B**) Representative image (Top) and histogram plot (bottom) shows western blot analysis of involucrin protein levels relative to β-actin in keratinocytes grown in control medium, hAMSCs-CM and hAECs-CM. Note: The values are expressed as means ±SEM. ****p < 0.0001; ***p < 0.001; **p < 0.01; *p < 0.05

We performed qRT-PCR analysis of differentiation markers to determine the effects of hAMSCs-CM and hAECs-CM on keratinocytes. The results showed that the expression of epidermal differentiation markers, involucrin, keratin 10, keratin 1, and filaggrin was significantly reduced in keratinocytes co-cultured with hAMSCs-CM and hAECs-CM compared to the controls (Figure 4A). Western blot analysis also showed that involucrin protein levels were significantly reduced in keratinocytes co-cultured with conditioned media for 3 days compared with the controls (Figure 4B). These data demonstrate that hAMSCs-CM and hAECs-CM inhibit keratinocyte differentiation.

### Mass spectrometry and bioinformatics analysis of hAMSCs-CM and hAECs-CM

**Figure 5:**
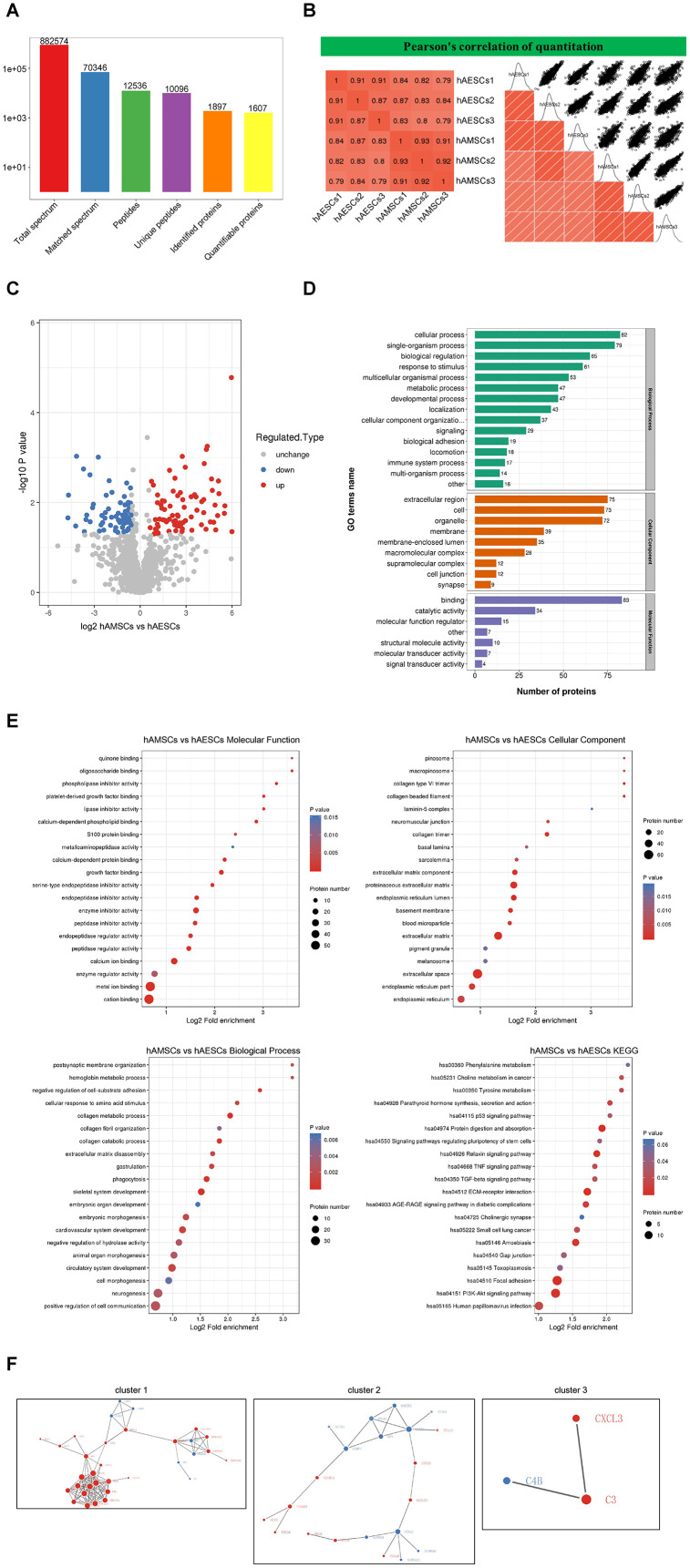
**Mass spectrometry and bioinformatic analyses of proteins in hAMSCs-CM (condition media of hAMSCs) and hAECs-CM (condition media of hAECs).** (**A**) Basic summary of mass spectrometric data. (**B**) A heat map compares Pearson correlation coefficients of proteins in hAMSCs-CM and hAECs-CM samples. A value closer to -1 indicates negative correlation, whereas a value closer to +1 indicates positive correlation. A value of 0 denotes no correlation. (**C**) Quantitative volcano map of differentially expressed proteins in conditioned media. The horizontal axis shows the Log_2_ relative expression value of the proteins, whereas, the vertical axis denotes the logarithmic p-value. The red dots indicate significantly up-regulated proteins and the blue dots indicate significantly down-regulated proteins. (**D**) Top differentially expressed proteins in the conditioned media classified according to three GO domains: biological process, molecular function and cellular component. (**E**) A directed acyclic graph shows GO and KEGG enrichment analyses of differentially expressed proteins in conditioned media. The circles indicate the GO classification of the differential expressed protein; the red color indicates highly significant protein (P<0.01), the yellow color indicates significant protein (P<0.05), and the blue color indicates no significance. The line with an arrow indicates the upper and lower levels of relationship according to the GO classification, and the circle size denotes the degree of enrichment. (**F**) The protein-protein interaction network map of the differentially expressed proteins in the conditioned media. The circles indicate differentially expressed proteins. The blue color denotes down-regulated proteins and the red color denotes up-regulated proteins. The size of the circle shows the number of proteins interacting with each other. A larger circle denotes a protein with several interacting partners.

Next, we used label-free mass spectrometry to analyze the proteins in hAMSCs-CM and hAECs-CM. We identified 1897 proteins in the conditioned media and quantitative information was available for 1607 proteins (Figure 5A). Pearson correlation analysis showed significant similarity in the composition and levels of proteins in hAMSCs-CM and hAECs-CM (Figure 5B). In comparison to hAECs-CM, hAMSCs-CM contained 70 downregulated and 84 upregulated proteins with a fold-change ≥1.5 and FDR <5% (Figure 5C). Next, we performed gene ontology (GO) and KEGG pathway analyses to determine the molecular functions, biological process, cellular component, subcellular localization, and signaling pathways represented by the proteins in hAECs-CM and hAMSCs-CM (Figure 5D). The bubble diagram shows the results of enrichment analysis using Fischer’s exact test to determine the GO functions and KEGG pathways that are significantly enriched (p<0.05; Figure 5E). Finally, we used the STRING (v.10.5) database to construct a protein interaction network that shows the relationship between these enriched proteins in hAECs-CM and hAMSCs-CM (Figure 5F).

### Both hAECs-CM and hAMSCs-CM contain high levels of proteins involved in skin development, ageing and wound healing

**Figure 6:**
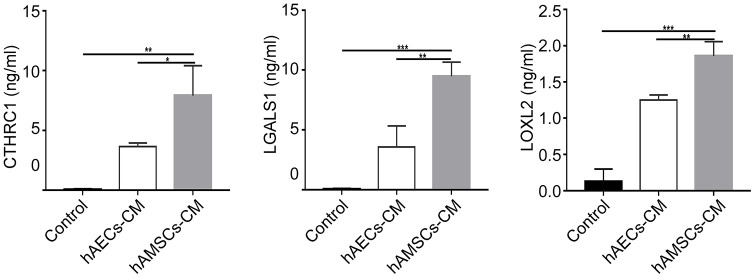
**The expression of CTHRC1 (Collagen triple helix repeat containing 1), LOXL2 (Lysyl oxidase-like 2) and LGALS1 (Galectin-1) protein in hAMSCs-CM (condition media of hAMSCs) and hAECs-CM (condition media of hAECs).** The histogram plots show the levels (ng/ml) of CTHRC1, LOXL2, and LGALS1 proteins in the control medium, hAECs-CM and hAMSCs-CM based on ELISA assays. The values are expressed as means ±SEM. ***p < 0.001; **p < 0.01; *p < 0.05.

The enrichment analysis showed that hAMSCs-CM contained high levels of proteins related to angiogenesis and inflammation (Table 1). The hAECs-CM was enriched with many proteins related to cell morphological development, skin formation, tissue remodeling, DNA damage repair, stress regulation and cell cycle (Table 2). Tables 1 and 2 lists differentially expressed proteins in the hAMSCs-CM and hAECs-CM that are responsible for wound healing, cellular differentiation, ageing, and cellular motility. Table 3 lists highly expressed proteins in the hAMSCs-CM that are related to wound healing, including those involved in epithelial morphogenesis and cell migration. These include CTHRC1, LOXL2, ADAMTS1, LGALS1, C3, and CYR61. Previous studies have reported the functions of CYR61 [28] and ADAMTS1 [29] in keratinocytes. We performed ELISA assay and confirmed that the levels of CTHRC1 [30, 31], LOXL2 and LGALS1 [32, 33] proteins were significantly higher in hAMSCs-CM compared to hAECs-CM (Figure 6).

**Table 1:** hAMSCs high-secreted proteins about wound healing, response to stimulus and cell development.

**GO Terms Description**	**GO Terms ID**		**Gene name**
**regulation of cell differentiation**	GO:0030154		SPON2, NRP1, LOXL2, HTRA1, GPC1, SERPINF1, COL4A1, MMP14, SPOCK1, MATN2, MAP1B, COL3A1, COL4A2, EXT1, COL12A1, SERPINE2, CYR61, B4GAT1, CTHRC1, MYH9, LGALS1, INHBA, SERPINH1, RPS7, CDH2, POSTN, VCAN, GLO1, COL6A1, EFEMP1
	GO:0048762		
	GO:0045597		
	GO:0045595		
	GO:0000904		
	GO:0045596		
**Wound healing**	GO:0042060		COL1A2, ANXA5, MYH9, MYL9, COL3A1, POSTN, TIMP1, PROCR, SERPINE2, CYR61, DCN, SERPINE1, CTHRC1, RPS7, NRP1, LGALS1, MATN2, MMP14, LOXL2, COL4A1, COL4A2, EXT1, COL12A1, INHBA, SERPINH1, CDH2, PLOD1, COL6A1, EFEMP1
	GO:0042061		
	GO:0042062		
	GO:0042063		
	GO:0042064		
	GO:0042065		
	GO:0042066		
	GO:0042067		
	GO:0042068		
**about aging**	GO:0042069		SOD2, TXNDC5, LOXL2, MYDGF, PEA15, MMP14, COL3A1, SERPINE2, TXNRD1, IGFBP4, CHID1, CXCL3, SERPINA3, LGALS3BP, MYH9, PDIA6, MYL9, INHBA, PDGFD, SERPINH1, C3, TIMP1, SERPINE1, PLOD1, DCN, SPON2, NRP1, SERPINB6, MATN2, SRPX, COTL1, LGALS1, IGFBP7, GLUL, POSTN, PDIA4, PLOD2, PROCR, SERPINF1, HTRA1
	GO:0042070		
	GO:0042071		
	GO:0042072		
**regulation of locomotion**	GO:0030336	GO:0051674	TIMP1, SRPX2, NRP1, LOXL2, MYH9, CXCL3, PDGFD, MMP14, POSTN, SERPINE2, CYR61, COL1A2, CTHRC1, GPC1, SPOCK1, MATN2, CDH2, VCAN, PROCR, INHBA, SPON2, EXT1, B4GAT1, GLO1, COL6A1, EFEMP1
	GO:0090132	GO:0010633	
	GO:0010596	GO:0016477	
	GO:2000145	GO:0010631	
	GO:0051270	GO:0030308	
	GO:0048870	GO:0040011	
	GO:0030335	GO:0010595	
	GO:0090130	GO:0030334	
	GO:2000147	GO:0040012	
	GO:0010594		
**cell growth and proliferation**	GO:0001558	GO:0048588	NRP1, MAP1B, IGFBP7, SERPINE2, IGFBP4, CYR61, POSTN, LGALS1, COL3A1, CTHRC1, RPS7, MYDGF, COL4A1, PDGFD, GLUL, TIMP1, LOXL2, GPC1, SERPINF1, COLQ, COL4A2, SRPX2, MYH9, EFEMP1, PPIB, C3, CDH2, SERPINE1, DCN, SRPX, CXCL3, P3H1, ADAMTS1, SOD2, B4GAT1, SPON2, MATN2, EXT1
	GO:0010721	GO:0048639	
	GO:0016331	GO:0030307	
	GO:0001936	GO:0050679	
	GO:0060562	GO:0051093	
	GO:0008284	GO:0048729	
	GO:0060485	GO:0048638	
	GO:0050793	GO:0002009	
	GO:0050673	GO:0000902	
	GO:0050678	GO:0016049	
	GO:0042127	GO:0051094	
	GO:0060284		
**inflammatory response**	GO:0001817	GO:0006954	CHID1, C3, PDGFD, CXCL3, MMP14, NRP1, SERPINF1, PTX3, SERPINA3, IGFBP4, MYH9, GLO1, COL1A2, GPC1, PROCR, LGALS1
	GO:0071675	GO:1902105	
	GO:0002687	GO:0050900	
	GO:0050921	GO:0002685	
	GO:0045577	GO:0051249	
	GO:0002688	GO:0050864	
	GO:0001819	GO:0050729	
	GO:0002690	GO:0050920	
**regulation of angiogenesis**	GO:1901343	GO:0060840	COL4A2, COL3A1, SRPX2, COL1A2, LOXL2, MYH9, COL4A1, MYDGF, MMP14, CDH2, LTBP1, CYR61, LGALS1, INHBA, GLO1, PDGFD, C3, ANXA5, MYL9, PROCR, SERPINE2
	GO:0048844	GO:0030097	
	GO:0001944	GO:1904018	
	GO:0016525	GO:0048514	
	GO:0001569	GO:0045766	
	GO:0043534	GO:0007599	
	GO:0001525		

**Table 2:** hAECs high-secreted proteins about wound healing, response to stimulus and cell development.

**GO Terms Description**	**GO Terms ID**		**Gene name**
**regulation of cell differentiation**	GO:0030855		ANXA4, MAP2K1, EZR, SFN, FLNB, EYA1
	GO:0030216		
	GO:0030217		
**regulation of wound healing**	GO:0030218		MAP2K1, GSN, ANXA2, MAP2K1
	GO:0030219		
**response to stimulus**	GO:0030220		TGM2, MAPK3, MAP2K1, C4B, ANXA2, FNTA, PSMB4, CHMP1A, KIF5B, SRI, HP, CLU, ANXA3
	GO:0030221		
	GO:0030222		
**regulation of blood circulation**	GO:1903522		SRI, GNAO1
**regulation of cellular localization**	GO:0060341		KIF5B, MAP2K1, EZR, NPEPPS, SFN, ANXA2, VAMP2, SCFD1, MAPK3, GSN, SRI, YWHAZ
	GO:0030223		
**regulation of cell cycle**	GO:1902806	GO:1903047	SFN, RPS27L, PSMD6, PSMB4, PSMB6, TUBA4A, EYA1, CHMP1A, MAP2K1, EZR, MAPK3, CLU, C4B
	GO:1901991	GO:2000134	
	GO:1902807	GO:0045786	
	GO:0000278	GO:0022402	
	GO:0007049	GO:0010948	
	GO:0044819	GO:1901987	
	GO:1902750	GO:0031571	
	GO:0016064	GO:1901988	
	GO:0000075	GO:0050852	
	GO:0044774	GO:0007093	
	GO:0010564	GO:0045930	
**inflammatory response**	GO:0002920	GO:0002520	HPX, C4B, MAPK3, MAP2K1, ADD1, ANXA2, TGM2, PSMB4, CLU, PSMD6, PSMB6, HP, AOC3, IL1RAP, ANXA3
	GO:0050727	GO:0002250	
	GO:0045089	GO:0031349	
	GO:0006954	GO:0009617	
	GO:0002253	GO:0002460	
	GO:0050778	GO:0019724	
**cell development**	GO:0002064	GO:0042246	EZR, SFN, FLNB, GSN, NACA, ADD1, KIF5B, S100A6, CLU, SCFD1, MAPK3, MAP2K1, LAMB3, LAMA3, LAMC2, ANXA3, ANXA4, EYA1, MINPP1, TGM2, S100A4, ANXA2
	GO:0048589	GO:0000902	
	GO:0040007	GO:0043588	
	GO:0045927	GO:0031099	
	GO:0008544	GO:0048639	
	GO:0003334	GO:0009888	
	GO:0009888	GO:0048146	
**about aging**	GO:0006281	GO:0080135	COPS5, RAD23B, RPS27L, EYA1, CLU, MAP2K1, HPX, EZR, PSMD6, ANXA2, PSMB4, TGM2, ERP29, MAPK3, GSN, C4B, PSMB6, NPEPPS, SFN, SCFD1, PDLIM1, GNAO1, HP
	GO:0080134	GO:0071453	
	GO:0006974	GO:0070482	
	GO:0036293	GO:2001020	
	GO:0007568	GO:2000377	
	GO:0036294	GO:0044773	

**Table 3:** hAMSCs high-secreted proteins positive biological phenomenon function in keratinocytes.

**GO Terms ID**	**GO Terms Level**	**GO Terms Description**	**Gene name**
GO:0016331	7	morphogenesis of embryonic epithelium	CTHRC1, RPS7
GO:0008284	6	positive regulation of cell proliferation	NRP1, CTHRC1, HTRA1, MYDGF, PDGFD, GLUL, TIMP1, CYR61
GO:0030154	5	cell differentiation	SPON2, NRP1, LOXL2, HTRA1, GPC1, SERPINF1, COL4A1, MMP14, SPOCK1, MATN2, MAP1B, COL3A1, COL4A2, EXT1, COL12A1, SERPINE2, CYR61, B4GAT1, CTHRC1, MYH9, LGALS1, INHBA, SERPINH1, RPS7, CDH2, POSTN, VCAN, GLO1, COL6A1
GO:0090132	5	epithelium migration	NRP1, LOXL2, MYH9
GO:0050793	4	regulation of developmental process	NRP1, LOXL2, GPC1, SERPINF1, MYDGF, COLQ, MMP14, SPOCK1, MAP1B, COL3A1, COL4A2, SERPINE2, CYR61, SRPX2, CTHRC1, MYH9, LGALS1, PDGFD, INHBA, EFEMP1, PPIB, C3, CDH2, POSTN, TIMP1, SERPINE1, DCN
GO:0042060	5	wound healing	COL1A2, ANXA5, MYH9, MYL9, PDGFD, COL3A1, POSTN, TIMP1, PROCR, SERPINE2, CYR61, DCN
GO:0002009	6	morphogenesis of an epithelium	NRP1, CTHRC1, COL4A1, MMP14, RPS7, CYR61
GO:0040017	5	positive regulation of locomotion	CXCL3, SRPX2, NRP1, POSTN, SERPINE1, PDGFD, MMP14, CYR61
GO:0010632	8	regulation of epithelial cell migration	SRPX2, NRP1, SERPINF1, DCN
GO:0045597	6	positive regulation of cell differentiation	NRP1, CTHRC1, LOXL2, GPC1, SERPINF1, INHBA, MMP14, CDH2, MAP1B, SERPINE2, CYR61
GO:0090130	4	tissue migration	NRP1, LOXL2, MYH9
GO:0060284	6	regulation of cell development	NRP1, LGALS1, SERPINF1, SPOCK1, CDH2, MAP1B, COL3A1, POSTN, SERPINE2
GO:1903034	6	regulation of response to wounding	SERPINE2
GO:0045595	5	regulation of cell differentiation	NRP1, LOXL2, GPC1, SERPINF1, MMP14, SPOCK1, MAP1B, COL3A1, SERPINE2, CYR61, CTHRC1, LGALS1, INHBA, EFEMP1, CDH2, POSTN
GO:0030307	7	positive regulation of cell growth	NRP1, MMP14, MAP1B
GO:0009611	4	response to wounding	COL1A2, ANXA5, NRP1, MYH9, LGALS1, MYL9, PDGFD, MATN2, COL3A1, POSTN, TIMP1, PROCR, SERPINE2, CYR61, DCN
GO:0010631	8	epithelial cell migration	NRP1, LOXL2, MYH9
GO:0048771	4	tissue remodeling	CTHRC1, MMP14
GO:0009888	4	tissue development	NRP1, LOXL2, COL4A1, MMP14, COL3A1, COL4A2, EXT1, COL12A1, SERPINE2, CYR61, CTHRC1, INHBA, SERPINH1, CDH2, RPS7, POSTN, PLOD1, TIMP1, COL6A1, DCN
GO:0040012	4	regulation of locomotion	CXCL3, SRPX2, NRP1, SERPINF1, PDGFD, MMP14, COL3A1, POSTN, SERPINE1, TIMP1, SERPINE2, CYR61, DCN

### LOXL2 promotes migration and differentiation of keratinocytes

**Figure 7:**
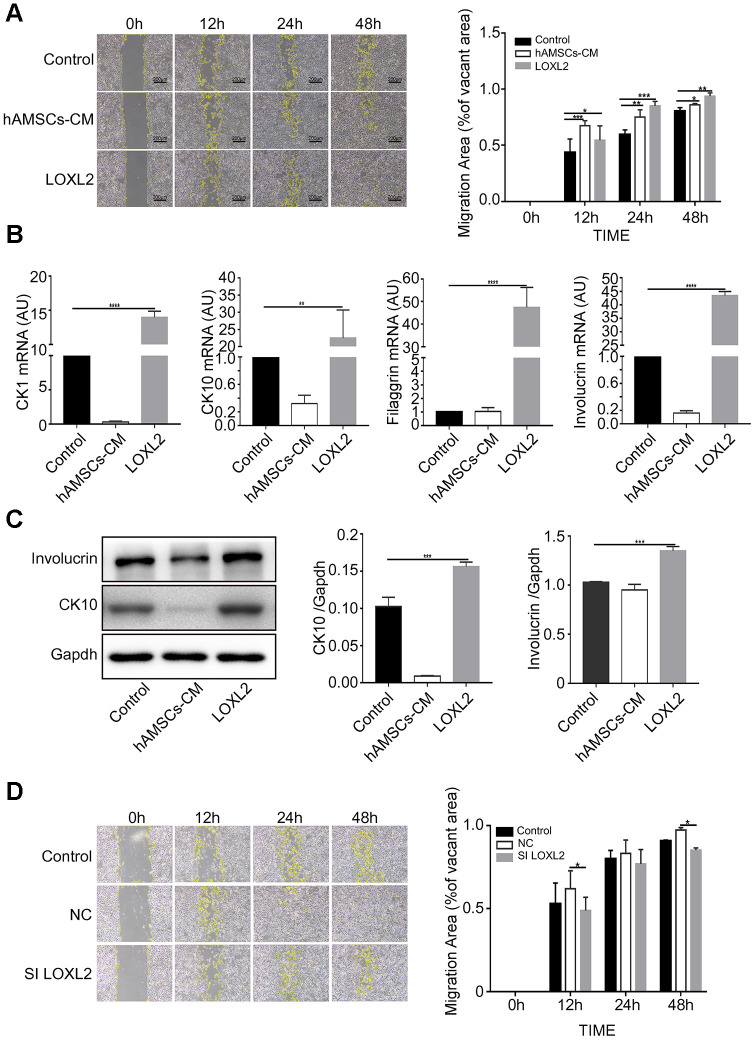
**LOXL2 (Lysyl oxidase-like 2) promotes migration and differentiation of keratinocytes.** (**A**) Representative images of the scratch wound assay show migration of keratinocytes at 0, 12, 24 and 48 h in control medium, LOXL2 and the hAMSCs-CM groups. The histogram shows the migration area in each group at various time points. (**B**) The histogram shows the relative mRNA levels of keratinocyte differentiation markers, CK1, CK10, Involucrin and Filaggrin in the control medium, LOXL2 and the hAMSCs-CM groups. (**C**) Representative images (left) and histogram plot (right) shows western blot results of involucrin and CK10 protein expression relative to GAPDH in the control medium, LOXL2 and the hAMSCs-CM groups. The values are expressed as means ±SEM. (**D**) Representative images show the results of the scratch wound assay at various time points (0, 12, 24 and 48 h) on the migration of keratinocytes in control medium, Si-NC-hAMSCs-CM and Si-LOXL2-hAMSCs-CM groups. The histogram plot shows the quantification of the cell migration area in each group at various time points. Note: ****p < 0.001; ***p < 0.001; **p < 0.01; *p < 0.05

We then performed scratch wound assay by co-culturing keratinocytes with 100 ng/ml LOXL2, CTHRC1 or LGALS1 for 3 days and observed that LOXL2 significantly increased keratinocyte migration compared to CTHRC1 or LGALS1 (Supplementary Figure 2). Furthermore, LOXL2 and hAMSCs-CM increased keratinocyte migration by similar levels (Figure 7A). Keratinocytes co-cultured with LOXL2 showed significantly higher expression of involucrin, keratin 10, keratin 1 and filaggrin mRNA compared to the controls (Figure 7B). Furthermore, western blot analysis showed that involucrin and keratin 10 protein levels were significantly higher in LOXL2-treated keratinocytes compared to the control (Figure 7C). This demonstrates that LOXL2 promotes keratinocyte differentiation. MTS assay showed that the cell proliferation rates were similar for LOXL-2 –treated and control keratinocytes (Supplementary Figure 3). Furthermore, to establish if LOXL2 was required for keratinocyte migration when incubated with hAMSCs-CM, we transfected hAMSCs with *LOXL2*-specific or control siRNAs for 24 h and then collected the condition medium (Supplementary Figure 4). Keratinocytes co-cultured with LOXL2-silenced hAMSCs-CM showed significantly reduced migration than those incubated NC-siRNA-hAMSCs-CM (Figure 7D). This demonstrates that LOXL2 protein in hAMSCs-CM promotes migration and differentiation of keratinocytes.

### LOXL2 promotes wound healing in mice

**Figure 8:**
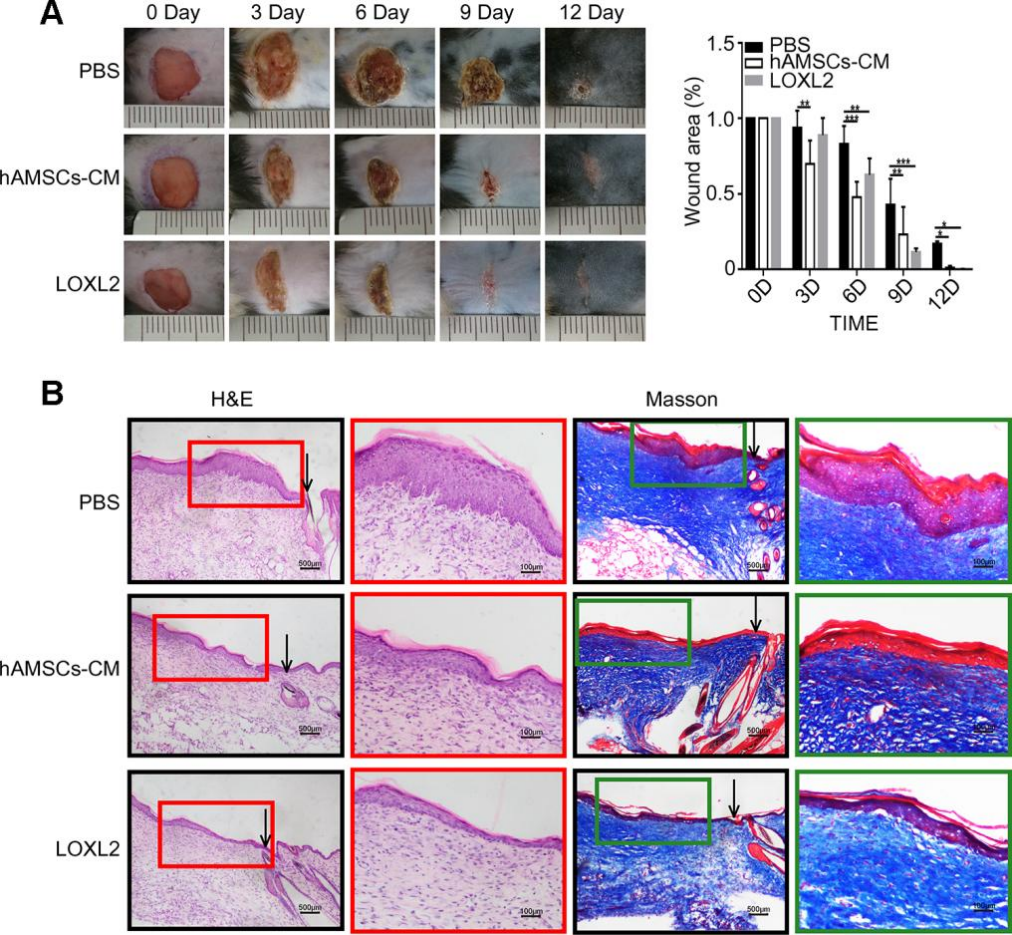
**LOXL2 (Lysyl oxidase-like 2) promoted wound healing in the mouse model.** (**A**) Representative photographs show the status of full-thickness excisional wounds in mice on days 0, 3, 6, 9 and 12. The wounds were treated with PBS (phosphate-buffered saline), 5X hAMSCs-CM (hAMSCs-CM concentrated five times), or LOXL2 (4ug). The histogram plot (right) shows the wound closure rate in the PBS, 5X hAMSCs-CM, and LOXL2 mice on days 0, 3, 6, 9 and 12. (**B**). Representative images (low and high resolution) show H&E (Hematoxylin and eosin staining) and Masson staining of wounded skin sections in mice belonging to PBS, 5X hAMSCs-CM and LOXL2 groups on day 14. Note: The values are shown as means ±SEM. ***p < 0.001; **p < 0.01; *p < 0.05.

Next, we investigated the *in vivo* effects of treatment with hAMSCs-CM and LOXL2 on wound closure using the mouse model of wound healing. None of the mice became sick or died during the treatments. The wound size in mice treated with hAMSCs-CM or LOXL2 were similar to that of the control on day 1 after treatment (data not shown). The lesion area was significantly reduced in the hAMSCs-CM group on day 3 and on day 6 in the LOXL2 group compared to the control group (Figure 8A). The wound sizes were significantly reduced in the LOXL2 and hAMSCs-CM groups on day 6 and completely healed by day 9 compared to complete healing on day 12 in the control group mice (Figure 8A).

Histological examination showed that hAMSCs-CM and LOXL2 enhanced wound healing compared to the control mice (Figure 8B). H&E staining of the skin sections showed that the epidermis of the hAMSCs-CM and LOXL2 group mice resembled normal skin and the keratinocytes were well organized and tightly arranged, whereas, the epidermal layer was thick and swollen and the keratinocytes were scattered in the control group mice (Figure 8B). Masson staining showed significantly reduced fibrosis and improved keratinocyte proliferation in the hAMSCs-CM and LOXL2 group mice compared to the control group mice (Figure 8B).

### JNK were involved in LOXL2 promoted keratinocyte migration and differentiation

**Figure 9:**
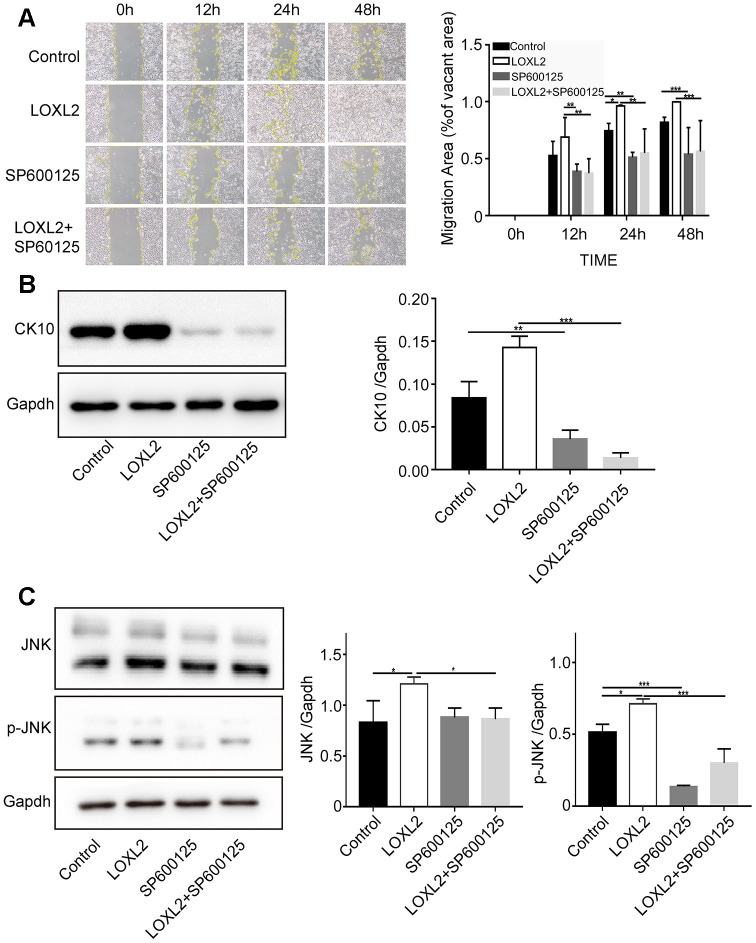
**LOXL2 (Lysyl oxidase-like 2) promotes keratinocyte migration and differentiation via JNK (c-Jun N-terminal kinase) signaling pathway.** (**A**) Representative images show the migration of keratinocytes based on the scratch wound assay in the control medium, LOXL2, SP600125 (JNK inhibitor), and SP600125 plus LOXL2 groups at various time points (0, 12, 24 and 48 h). The histogram plot shows the cell migration area in each group at various time points. (**B**) Representative image (left) and histogram plot (right) shows the expression of CK10 protein in the keratinocytes belonging to control medium, LOXL2, SP600125 and SP600125 plus LOXL2 groups. GAPDH was used as loading control. The values are expressed as means ±SEM. (**C**) Representative image (left) and histogram plot (right) shows the levels of JNK and phospho-JNK in the keratinocytes from control medium, LOXL2, SP600125 and SP600125 plus LOXL2 groups. The values are expressed as means ±SEM. ***p < 0.001; **p < 0.01; *p < 0.05.

Previous studies and KEGG pathway analysis (ko04010) suggest that LOXL2 regulates migration and differentiation of keratinocytes via the JNK pathway [34–36]. Therefore we analyzed migration and differentiation of keratinocytes after preincubating them with 10μM JNK inhibitor SP600125 for 1h followed by co-culture with LOXL2. Scratch wound assay results showed that treatment with 10μM SP600125 significantly reduced keratinocyte migration (Figure 9A). Western blot analysis showed that the expression of keratin 10 protein was significantly reduced in keratinocytes preincubated with 10μM SP600125 for 24h compared to the control (Figure 9B). Furthermore, western blot analysis showed that LOXL2 treatment significantly increased JNK phosphorylation in the keratinocytes, whereas, preincubation with 10μM SP600125 significantly reduced JNK phosphorylation compared to the controls (Figure 9C). These results show that LOXL2 promotes wound healing through keratinocyte migration and differentiation via the JNK signaling pathway.

## DISCUSSION

The cells derived from human amniotic membranes (hAM) are suitable for regenerative medicine because they are associated with low immunogenicity and possess anti-inflammatory, antifibrotic, and other clinically relevant properties [37, 38]. Dehydrated human amnion/chorion membrane (DHCM) allografts significantly enhance wound healing and rectify full-thickness scalp and lower eyelid defects after Mohs micrographic surgery [39, 40]. Murphy et al. showed that amnion membrane-derived hydrogel and amnion membrane powder significantly enhanced the formation of a mature epidermis in the mouse wound healing model and a full-thickness porcine skin wound healing model [41]. Moreover, dehydrated human amniotic membranes and radiation-sterilized amnion dressings are highly effective in healing surgical wounds [42, 43]. Insausti et al., showed that the amniotic membrane (AM) wound dressing restored skin integrity in patients with deep and traumatic wounds and helped avoid the need for skin graft reconstruction [44]. Castellanos et al*.,* showed that AM promotes proliferation and migration of keratinocytes by antagonizing the effects of TGF-β, thereby promoting epithelialization of chronic wounds [45]. Zheng et al. showed that transplantation of cryopreserved living micronized amnion onto the wounds of diabetic mice regulates macrophage migration and phenotype switching and increases neovascularization [46]. Koob et al., showed that angiogenic cytokines such as angiopoietin-2 (ANG-2), epidermal growth factor (EGF), basic fibroblast growth factor (bFGF), heparin binding epidermal growth factor (HB-EGF), hepatocyte growth factor (HGF), platelet derived growth factor BB (PDGF-BB), and placental growth factor (PlGF) in dehydrated amnion/chorion grafts induce endothelial cell proliferation and support the formation of blood vessels [47]. Koob et al*.* also showed the presence of platelet-derived growth factor-AA (PDGF-AA), PDGF-BB, transforming growth factor α (TGFα), TGFβ1, basic fibroblast growth factor (bFGF), epidermal growth factor (EGF), placental growth factor (PLGF) and granulocyte colony-stimulating factor (GCSF) in the DHACM allografts that promote wound healing by inducing cell proliferation, inflammation, metalloproteinase activity and recruitment of progenitor cells to the wound sites [48]. Moreover, amniotic membrane products contain paracrine factors that shorten wound healing times and reduce pain [49].

The human amniotic membrane consists of hAECs from the ectoderm and hAMSCs from the mesoderm. Both cell types produce the extracellular matrix (ECM), cytokines and other growth factors [37, 50]. In this study, we demonstrate that the conditioned medium from hAECs and hAMSCs cultures have similar composition and characteristics. *In vivo* studies show that both hAECs and hAMSCs possess anti-inflammatory and tissue remodeling properties and are effective in healing acute and chronic skin wounds [50]. AMSCs promote endothelial cell proliferation [51] and angiogenesis [52], whereas, exosomes derived from hAECs inhibit scar formation [53]. We previously showed that hAMSCs accelerate wound healing and re-epithelialization in the rat wound healing model [54]. Previous studies have shown that hAECs-CM or exosomes derived from hAECs induce keratinocyte migration and accelerate wound healing [53, 55].

In this study, we investigated the biological effects of hAECs-CM and hAMSCs-CM on the proliferation, migration, and differentiation of keratinocytes and their subsequent role in wound healing. Our data shows that both hAMSCs-CM and hAECs-CM induced migration of keratinocytes in the scratch wound assay. Keratinocyte migration was slightly higher in the hAMSCs-CM compared to the hAECs-CM. Moreover, hAMSCs-CM inhibits keratinocyte proliferation more strongly than hAECs-CM. However, these results contradict a previous study that showed hAECs-CM promoting keratinocyte proliferation [11]. The contradictory results could be because of the different cell types used in both studies (keratinocytes derived from foreskin vs. HACAT cell line) and the differences in the basic serum-free culture medium used. Further studies are needed to dissect the differences between the two types of cells.

Our study shows that hAMSCs-CM have a stronger ability to inhibit keratinocyte differentiation and maintain stemness. Li et al*.* generated bilayered tissue-engineered skin by growing hAECs on fibrin scaffolds and using hAMSCs to induce proliferation and differentiation of the hAECs and form skin-like layers of keratinocytes [56]. This shows that hAMSCs secrete proteins that promote growth and development of the epidermis. Cell apoptosis was observed when hAECs were continuously cultured after day 3 in MTS assy (The Supplementary Figure 1C). hAECs-CM is hard to maintain the development of keratinocytes in vitro for a long time when keratinocyte proliferation was significantly inhibited. In this study, we observed that both hAMSCs-CM and hAECs-CM promote migration of keratinocytes, although hAECs-CM was slightly stronger than hAMSCs-CM. However, hAMSCs are more genetically stable for large-scale clinical applications and product development. The hAMSCs can proliferate up to 30 passages compared to 5 passages for the hAECs, which show an early tendency to differentiate; hAMSCs are also easier to culture and freeze *in vitro*, and are more suitable for large-scale cultures and to collect conditioned media [12, 57, 58]. The hAMSCs also possess strong anti-inflammatory properties and help maintain optimal development of the epidermis in the wound microenvironment by promoting the proliferation, migration, and differentiation of the keratinocytes [59]. Therefore, we analyzed the proteins that are secreted by the hAMSCs and hAECs into the conditioned media that are amenable for future clinical applications, including treatment of various wounds and skin disorders.

As shown in Table 2, hAMSCs-CM is enriched with proteins that regulate wound healing, angiogenesis, cell differentiation, immune response and cell motility. Interestingly, hAECs-CM promotes migration of keratinocytes better than hAMSCs-CM. One reason for this is that both hAECs and keratinocytes are derived from ectodermal cells and hence, probably secrete more homologous chemokines and growth factors [55]. The hAMSCs secrete proteins that also promote migration of keratinocytes. The results of GO analysis (Supplementary Table 1) show that hAMSCs-CM proteins may regulate neurogenesis, angiogenesis, organ morphogenesis, and skeletal system development. Injection of hAMSCs into rats with intracerebral hemorrhage and traumatic spinal cord injury promotes recovery of neurological function by inhibiting inflammation and apoptosis, and promoting neurogenesis and angiogenesis [60–62]. Moreover, hAMSCs promote osteogenic differentiation of human bone marrow mesenchymal stem cells (hBMSCs) and human adipose-derived stem cells (hADSCs) [63, 64]. They ameliorate oxidative stress-induced dysfunction of the human BMSCs [65]. The extracellular vesicles derived from hAMSCs suppress inflammation and fibrosis in the rat model of nonalcoholic steatohepatitis [66]. The hAMSCs inhibit natural ovarian ageing by secreting factors such as hepatocyte growth factor and epidermal growth factor [67]. These reports confirm that hAMSCs secrete proteins and exosomes that inhibit inflammation and organ ageing, promote angiogenesis, neurogenesis, and osteogenic differentiation, and protect organ functions. These results are consistent with our mass spectrometry data.

The lysyl oxidase (LOX) family of enzymes including LOXL2 catalyzes the cross-linking of extracellular matrix proteins, collagen and elastin [68]. LOXL2 also regulates several extracellular and intracellular TGF-β andPI3K / Akt / mTOR cell signaling pathways [19, 69]. LOXL2 gene expression is observed in the heart, placenta, testis, lung, kidney, uterus, brain and the liver [19]. LOX proteins also regulate development, senescence, tumor suppression, cellular growth, and chemotaxis [70, 71]. Chvapil et al. first reported the role of LOX proteins in the wound healing process [72]. LOXL2 cross-links lysine residues in extracellular matrix proteins and maintains the structural integrity of the connective tissues [20]. The *in vivo* expression of LOXL2 is significantly increased during the cartilage formation stage of healing fractures [23, 73]. LOXL2 relieves osteoarthritis by promoting a chondroprotective response that includes, induction of anabolic gene expression, remodeling of the extracellular matrix and attenuation of the expression of catabolic genes [74]. LOXL2 expression is induced in the mouse model of choroidal neovascularization and its inhibition significantly reduces angiogenesis and inflammation [75]. LOXL2 is expressed as a hypoxic target in the angiogenic endothelial cells, and its accumulation in the extracellular matrix regulates angiogenesis [22]. The intra- and extra-cellular functions of LOX proteins play a significant role in the normal ageing process of skin as well as several skin disorders [76]. LOXL2 plays an important role in gene expression that regulates several intra-and extracellular functions related to wound healing [77]. Our study demonstrates high LOXL2 concentration in the hAMSCs-CM. Furthermore, we demonstrate that LOXL2 promotes migration and differentiation of keratinocytes, which is required for re-epithelialization.

In conclusion, our study demonstrates that conditioned medium derived from hAMSCs and hAECs contain proteins that promote wound healing both *in vitro* and *in vivo*. This includes LOXL2, which promotes wound healing by activating the JNK signaling pathway. Our study suggests potential clinical applications for LOXL2 in skin re-epithelialization and wound healing. Furthermore, clear proteins would achieve more effective targeted treatment than conditioned medium. It also reduces ethical arguments and constraints on product development caused by insufficient amniotic tissue sources.

## MATERIALS AND METHODS

### Isolation and culturing of hAMSCs and hAECs

The human amniotic epithelial stem cells (hAECs) were isolated and cultured as previously reported [11]. Then, the amnion membrane was cut into small pieces and digested with 1mg/ml Collagenase type IV (Sigma, USA) for 1h. The cell suspension was centrifuged to pellet down the human amnion mesenchymal stem cells (hAMSCs). The hAMSCs were cultured in DMEM-F12 (Hyclone, USA) medium supplemented with 10% fetal bovine serum (Hyclone, USA), 100 U/mL penicillin, and 100 ng/mL streptomycin (Gibco, USA) at 37°C and 5% CO2. To obtained the high purity of hAECs, we prepared conditioned media in passages 3.

### Preparation of conditioned media

When hAECs and hAMSCs reached 90 % confluence in passage 2, 2.5×10^6^ cells were seeded into 75cm² cell culture flasks in serum-free EpiLife medium for 24h. The culture medium was collected from each sample and used as conditioned media (CM).

### Isolation and culturing of keratinocytes

We cut 2cm strips human foreskin samples, removed subcutaneous fat, and treated them overnight with 3mg/ml Dispase II (Sigma, USA) at 4°C. Then, the epidermis was separated, cut into small pieces and digested with Trypsin/EDTA solution (Biological Industries, IL, USA) for 30 min at 37°C. The trypsin digestion was stopped by adding bovine serum (Hyclone, USA) and the cell suspension was strained through a 40 μm filter to filter out any debris. The cell suspension was centrifuged to pellet the keratinocytes and cultured in EpiLife medium (Gibco; USA) supplemented with human keratinocyte growth supplement (HKGS, Gibco; USA) at 37°C and 5% CO_2_. The keratinocytes were cultured in 6-well plates until they reached 40% confluence. Then, we added 200 nanograms of LOXL2 (Sino Biological, China), CTHRC1 (Novoprotein, China) or LGASL1 (Novoprotein, China) to the wells and the keratinocytes were further cultured for 3 days and analyzed.

### Flow cytometry analysis of hAMSCs and hAECs

The hAMSCs and hAECs were stained with phycoerythrin(PE)-conjugated antibodies against CD73, CD45, CD31, CD105, CD44, CD34, CD90, CD29, SSEA-3, SSEA-4, EP-CAM, HLA-DR (Biolegend Pharmingen, USA) and analyzed using the flow cytometer with appropriate controls.

### Differentiation of hAMSCs and hAECs into osteoblasts, chondrocytes and adipocytes

We differentiated hAMSCs and hAECs into osteoblasts by growing them in human osteogenic differentiation medium (Cyagen, China) containing ascorbate, dexamethasone and FBS for 3 weeks. The cells were then fixed with 4 % formaldehyde and stained with Alizarin Red. We differentiated hAMSCs and hAECs into chondrocytes by growing them in human chondrogenic differentiation medium (Cyagen, China) containing dexamethasone, ascorbate, ITS supplement, sodium pyruvate, proline and TGF-β3 for 4 weeks. Then, the cells were fixed with 4 % formaldehyde and stained with Alcian Blue. We differentiated hAMSCs and hAECs into adipocytes by growing them in human adipogenic differentiation medium (MesenCult, Stem cell, CAN) containing adipogenic differentiation supplement and FBS for 3 weeks. The cells were then fixed with 4 % formaldehyde and stained with the Oil Red O.

### MTS cell proliferation assay

To analyze the effects of hAMSCs-CM and hAECs-CM on keratinocyte proliferation, we seeded 2.5×10^4^ keratinocytes per well in 96-well plates for 24h at 37°C and 5% CO2. Then, we added hAMSCs-CM or hAECs-CM. We added 10 μl MTS solution (Promega, USA) at 24, 48, or 72 h after adding conditioned media and further incubated the cells at 37°C for 2 h. The absorbance was measured at a wavelength of 450 nm in a colorimeter and their proliferation rate was quantified.

### Cell cycle analysis by flow cytometry

To analyze cell cycle analysis by flow cytometry, we seeded keratinocytes in 6-well plates until they reached 40% confluence. Then, hAMSCs-CM or hAECs-CM was added and grown for an additional 24 h. Then, the keratinocytes were trypsinized, washed with ice-cold PBS, and fixed in ice-cold 70% ethanol overnight. Then, after washing the cells once with ice-cold PBS, the cells were incubated in propidium iodide staining solution for 30min in the dark at 37°C. The cells were then immediately analyzed by flow cytometry and the percentages of G1, S and G2M phase cells in different samples were determined.

### Scratch wound assay

We performed the scratch wound healing assay to determine the effects of hAECs-CM and hAMSCs-CM on *in vitro* cell migration. We grew keratinocytes in 6-well culture plates until they reached 90% confluence. Then, we used a sterile pipette tip to generate a 500μm wide cell-free scratch and removed the non-adherent cells by washing twice with phosphate-buffered saline (PBS). We then added hAECs-CM, hAMSCs-CM or normal culture medium and photographed the cultures at 0, 12, 24, 36 and 48 h using a digital camera. We used the Image J software to analyze the width of the scratch area at different time points to determine the rate of migration of the keratinocytes in each well.

### Quantitative real-time reverse transcription polymerase chain reaction (qRT-PCR)

We performed qRT-PCR as previously reported [11]. The qPCR reaction was performed in a ABI 7500 Real-Time System using the SYBR1 Premix Ex Taq II kit (TaKaRa, China). The primers used for the qRT-PCR are shown in Table 4.

**Table 4:** Primers used in quantitative real time-PCR experiments and cell transfection assay.

	**Forward**	**Reverse**
**CK1**	TCATCAACTACCAGCGCAGG	ACCATAACCACCACCAAAGC
**CK10**	AGGAGGAGTGTCATCCCTAAG	AAGCTGCCTCCATAACTCCC
**Involucrin**	TCCTCCAGTCAATACCCATCAG	CAGCAGTCATGTGCTTTTCCT
**Filaggrin**	CAATCAGGCACTCATCACAC	ACTGTTAGTGACCTGACTACC
**GAPDH**	ACCACAGTCCATGCCATCAC	TCCACCACCCTGTTGCTGTA
**LOXL2-homo-1957**	GGAGUUGCCUGCUCAGAAATT	UUUCUGAGCAGGCAACUCCTT
**LOXL2-homo-590**	GCGAUGACGACUUCUCCAUTT	AUGGAGAAGUCGUCAUCGCTT
**LOXL2-homo-861**	CCAGAUAGAGAACCUGAAUTT	AUUCAGGUUCUCUAUCUGGTT

### Western Blot analysis

We separated 20 μg total protein lysate on a 10 % polyacrylamide gel. The separated proteins were transferred onto a polyvinylidene difluoride (PVDF) membrane. The membranes were blocked in 5% skim milk for 1 h by rocking gently. Then, the membranes were incubated overnight at 4 °C with rabbit anti-human LOXL2 antibody (Abcam, USA). The membranes were then washed thrice with PBST and incubated with the secondary horseradish-peroxidase-conjugated anti-rabbit antibody (Boster, China) at room temperature for 2 h. The protein bands were developed and visualized using enhanced chemiluminescent imaging system (Tanon, China).

### ELISA assay

We measured the levels of CTHRC1, LGASL1, and LOXL2 proteins in the conditioned medium using ELISA assay with commercially available kits (MLBIO, Shanghai, China). Briefly, 50 μl of conditioned medium was incubated with the detection antibody in the ELISA plate for 1 h at 37° C. After washing the plate, we added streptavidin and incubated the samples for 30 mins at 37°C. Then, after washing, we added the tetramethylbenzidine (TMB) susbtrate to the samples, incubated the plate for 10 minutes at 37°C, and added the stop buffer. The samples were analyzed by quantifying the colorimetric reaction at 450 nm in a colorimeter.

### *In vivo* wound healing mouse model

The wound healing mouse model protocol was approved by the Medical Ethics Committee of the China medical university, Shenyang, China. We obtained 6-week-old male C57 mice (20 ± 2 g) from the China medical University Laboratory Animal Center (CMU2019235) and housed them under standard laboratory conditions with a 12 h light/12 h dark cycle at 25 °C and provided a daily supply of food and water. We randomly divided the mice into three groups (n = 4): (1) phosphate-buffered saline (PBS) control group; (2) hAMSCs-CM treatment group (where the hAMSCs-CM was concentrated five times); and (3) murine LOXL2 (5μg, Sino Biologicals, China) treatment group. The mice were first anesthetized by intraperitoneal (i.p.) injection of pentobarbital sodium (20 mg/kg body weight.). The dorsal skin was shaved with an electric clipper and the skin was disinfected with 75% alcohol. Then, circular pieces (1cm) of full-thickness skin was cut off from pre-determined areas on the back of the mice using a sterile biopsy punch. Then, the mice were treated by injecting the surround tissues of the wound bed at four sites per mouse with 50μl PBS or 50μl hAMSCs-CM (5X) or 50μl LOXL2 (5μg). The wound area was analyzed on days 0, 3, 6, 9 and 12 using the Image J software (NIH, USA) and the percentage of wound contraction was determined using the following equation: Percent wound contraction = [(wound area on day 0 − wound area on a particular day)/ wound area on day 0] × 100. The mice were anesthetized on day 14 by injecting pentobarbital sodium (20 mg/kg body weight) intraperitoneally and euthanized by CO2 asphyxiation. The skin tissue samples were collected for further histological analysis and the wound area was calculated using the Image J software.

### Histological examination

The wounded skin tissues were collected from all groups of mice on day 14, fixed in 4% paraformaldehyde and dehydrated using a series of sucrose solutions before being immersed in the Optimum Cutting Temperature Compound (Sakura, USA). Then, the skin samples were embedded and cut into 8-μm-thick slices using the freezing microtome (Leica, GER). The tissue slices were mounted on the glass slides and stained with hematoxylin-eosin (H&E) and Masson stains (Solarbio, China) according to manufacturer’s instructions. The stained sections were photographed using an OlympusFSX100 microscope (Olympus, Japan).

### Mass Spectroscopy (MS/MS) analysis

The amnion tissues were obtained from 3 women that underwent caesarean deliveries and isolated hAMSCs and hAECs from the tissues as previously described (Isolation and culture of hAMSCs and hAECs). We collected biological duplicates of hAECs-CM and hAMSCs-CM for MS analysis by centrifugation at 12,000 g at 4 °C for 10 min. Then, the hAECs-CM and hAMSCs-CM were concentrated by centrifuging in an Amicon Ultra-50 Ultracel-3k centrifuge tube (Merck Millipore, DE) at 5000 xg at 4 °C for 2h. Finally, the protein concentration was determined using a BCA kit according to the manufacturer's instructions. Then, the concentrated protein solution was treated with 5 mM dithiothreitol for 30 min at 56 °C and alkylated with 11 mM iodoacetamide for 15 min at room temperature in darkness. The modified protein samples were then diluted by adding 100 mM Triethylammonium bicarbonate (TEAB) buffer and urea (concentration less than 2M). Finally, the samples were first digested overnight with trypsin at a 1:50 trypsin-to-protein mass ratio and again with a 1:100 trypsin-to-protein mass ratio for 4 h.

The trypsinized peptides were fractionated in a Thermo Betasil C18 column (5 μm particles, 4.6 mm ID, 250 mm length) using high pH reverse-phase HPLC. Briefly, the peptides were first separated into 60 fractions in an 8% to 32% acetonitrile (pH 9.0) gradient for 60 min. Then, the peptides were combined into 4 fractions and dried using vacuum centrifugation. The powdered tryptic peptides were then dissolved in 0.1% formic acid (solvent A) and directly loaded onto a home-made reverse-phase analytical column (15-cm length, 75 μm i.d.) and then fractionated in a EASY-nLC 1200 UPLC system using 9% to 22% solvent B (0.1% formic acid in 98% acetonitrile) gradient for 32 min, 22% to 35% gradient for 20 min, 35% to 80% gradient for 4 min and then at 80% gradient for the last 4 min, all at a constant flow rate of 350 nL/min.

The peptides were then subjected to nanospray ionization (NSI) followed by tandem mass spectrometry (MS/MS) in an Orbitrap Fusion Lumos Mass Spectrometer (Thermo, USA), which was coupled online to a UPLC at an electrospray voltage of 2.0 kV. The m/z scan range was 350 to 1550. Intact peptides were detected in the Orbitrap at a resolution of 60,000. The peptides were then selected for MS/MS using normalized collision energy (NCE) setting as 28, and the fragments were detected in the Orbitrap at a resolution of 15,000. The data-dependent procedure alternated between one MS scan followed by 20 MS/MS scans with a 30.0s dynamic exclusion. The automatic gain control (AGC) was set at 5E4 and the fixed first mass was set as 100 m/z.

The resulting MS/MS data was processed using the Maxquant search engine (v.1.5.2.8 http://www.maxquant.org/). The tandem mass spectra was searched against the *Human_SwissProt_1808* database (20387 sequences) and concatenated with the reverse decoy database. Trypsin/P was specified as cleavage enzyme allowing up to 2 missing cleavages. The mass error tolerance for precursor ions was set at 20 ppm in the first search and 5 ppm in the main search, and the mass error tolerance for fragment ions was set at 0.02 Da. The carbamidomethyl on Cys was specified as a fixed modification, and the acetylation on protein N-terminus and oxidation on Met were specified as variable modifications. FDR was adjusted to < 1% (PTM Biolab LLC, China).

### Bioinformatic analysis

The proteins in the hAMSCs-CM and hAECs-CM were processed using Maxquant search engine (v.1.5.2.8). Tandem mass spectra were searched against human UniProt database (20387 sequences, 201808) concatenated with reverse decoy database and commonly occurring contaminant. The proteins were annotated according to three Gene Ontology (GO) categories: biological process, cellular component and molecular function. The KEGG database (v.2.0 http://www.genome.jp/kaas-bin/kaas_main) was used to annotate the CM proteins to their corresponding signaling pathways. The WoLF_PSORT database (v.0.2 http://www.genscript.com/psort/wolf_psort.html) was used to predict the subcellular localization of the CM proteins. Then, the proteins in each functional category were filtered based on their differential expression using P value <0.05 as a filter. A protein-protein interaction network was constructed for all differentially expressed proteins using the STRING database version 10.1. Only interactions with a confidence score ≥ 0.7 (high confidence) were included. The interaction network was visualized using the R package “networkD3”.

### Statistical analysis

Statistical analysis was performed using the Graphpad Prism 7 software. The data are represented as the means ± standard error (S.E.) of at least three independent experiments. The samples were compared using the one-way ANOVA or Student’s t tests and a P value < 0.05 was considered statistically significant.

## Supplementary Material

Supplementary Methods

Supplementary Figures

Supplementary Table 1
