# Supplementary material for: LOXL2 from human amniotic mesenchymal stem cells accelerates wound epithelialization by promoting differentiation and migration of keratinocytes
Source: Aging (Albany NY). 2020 Jul 4;12(13):12960–86. doi: 10.18632/aging.103384 (PMC7377892; doi:10.18632/aging.103384)
Supplement: Supplementary Methods [file aging-12-103384-s001..pdf]

SUPPLEMENTARY METHODS

LOXL2 silencing decreases LOXL2 levels in hAMSC-CM

To further confirm that LOXL2 in hAMSC-CM promotes migration of keratinocytes, we silenced *LOXL2* gene expression using LOXL2-specific siRNAs. All three siRNAs downregulated *LOXL2* expression (Supplementary Figure 4A). The culture medium was changed at 24h after siRNA transfections and replaced with EpiLife medium and the cells were cultured and tested at 0, 6 and 24 h. Then, the conditioned medium was collected from all groups for the ELISA assay. The si-861 group showed the highest suppression of LOXL2 secretion into CM compared with the si-1957 and si-590 groups (Supplementary Figure 4B). ELISA assay shows LOXL2 protein levels were significantly reduced in the si-861-hAMSCs compared to the control hAMSC-CM (Supplementary Figure 4C). Hence, si-861 was used for further analysis.

Conditioned medium dilutions

After collecting the conditioned medium as previously described in the methods section, Epilife plus HCKS medium (GIBCO, USA) was added to obtain 0.25, 05, and 0.75 CM. For example, 0.25 CM or 25% CM was prepared by mixing 1ml condition medium to 3ml Epilife medium.

LOXL2 knockdown

We transfected hAMSCs (passage 3 and 90% confluent) with 10 nM si*LOXL2* or negative control (NC) using Lipofectamine 3000 (Thermo Fisher, USA) according to the manufacturer's instructions. All LOXL2-specific and control siRNAs were purchased from GenePharma (Shanghai, China). After 24 h, the medium was replaced with DMEM-F12 plus 10%FBS. After a defined period of time (0h, 6h, 24h), the cells were washed with PBS once and grown in EpiLife medium for 24h. Then, the culture medium was obtained from all samples and used as conditioned medium for further experiments.
