# Supplementary material for: LOXL2 from human amniotic mesenchymal stem cells accelerates wound epithelialization by promoting differentiation and migration of keratinocytes
Source: Aging (Albany NY). 2020 Jul 4;12(13):12960–86. doi: 10.18632/aging.103384 (PMC7377892; doi:10.18632/aging.103384)
Supplement: Supplementary Figures [file aging-12-103384-s002..pdf]

SUPPLEMENTARY FIGURES

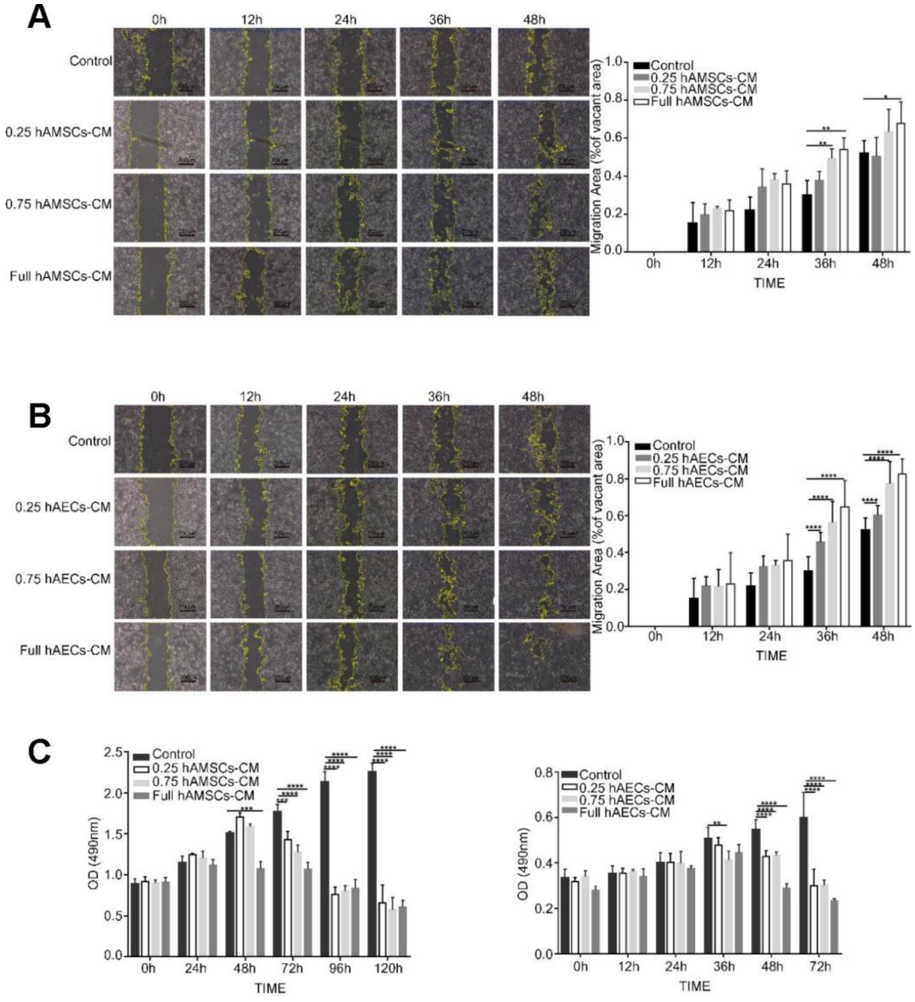

Supplementary Figure 1. Culturing with increasing proportion of hAMSCs-CM and hAECs-CM inhibits proliferation, but promotes migration of keratinocytes. (**A**) Representative images of the scratch wound assay show the migration of keratinocytes at various time points (0, 12, 24, 36, and 48 h) in different dilutions of hAMSC-CM. The histogram plot shows the quantification of the keratinocyte migration at different time points in all experimental groups. (**B**) Representative images of the scratch wound assay show the migration of keratinocytes at various time points in the different dilutions of hAEC-CM. The histogram plot shows the quantification of the keratinocyte migration at different time points in all experimental groups. (**C**) Histogram plot shows the results of MTS cell proliferation assay of keratinocytes grown in different dilutions of hAMSC-CM and hAEC-CM at 0, 24, 48, 72, 96 and 120 h. The values are shown as means ±SEM. ****$p < 0.0001$;***$p < 0.001$; **$p < 0.01$; *$p < 0.05$.

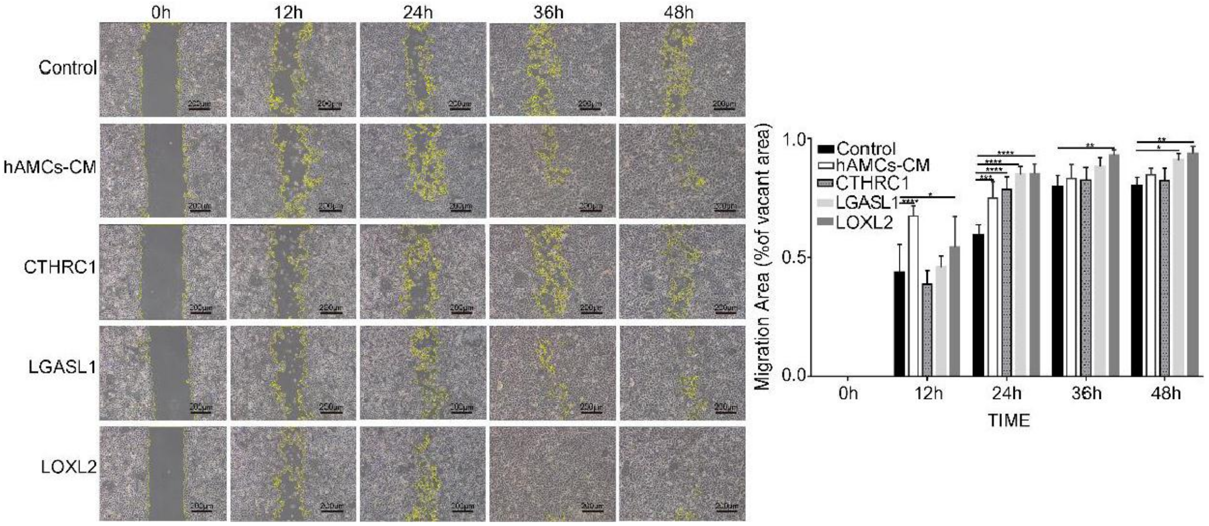

Supplementary Figure 2. Representative images of the scratch wound assay show the migration of keratinocytes at various time points (0, 12, 24, 36, and 48 h) in control medium, hAMSCs-CM, CTHRC1, LGASL1 and the LOXL2 groups. The histogram plot (right) shows the quantification of keratinocyte migration at various time points in all experimental groups. The values are shown as means ±SEM. ****p < 0.0001;***p < 0.001; **p < 0.01; *p < 0.05

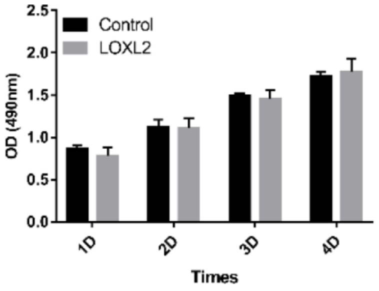

Supplementary Figure 3. The histogram plot shows the results of MTS cell proliferation assay in the control and LOXL2 treatment groups. The values are shown as means ±SEM.

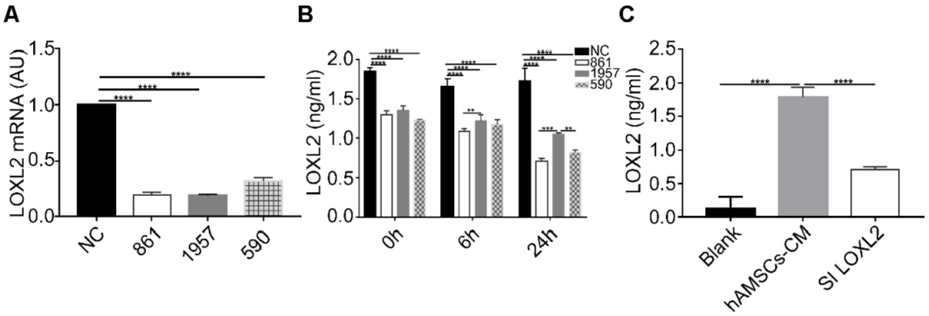

Supplementary Figure 4. LOXL2 silencing significantly reduces LOXL2 protein levels in hAMSC-CM. (**A**) The histogram plot shows relative LOXL2 mRNA levels from hAMSCs transfected with si-NC, si-861, si-1957, and si-590 for 24 h. (**B**) ELISA assay results show LOXL2 protein levels (ng/ml) in the conditioned media obtained from hAMSCs transfected with si-NC, si-861, si-1957, and si-590. The LOXL2 levels were measured at 0h, 6h and 24h after transfections. (**C**) ELISA assay results show LOXL2 levels in blank (EpiLife medium plus HKCs and without any treatment), hAMSC-CM, and si-861-hAMSC-CM. The values are means ±SEM. ****p < 0.0001;***p < 0.001; **p < 0.01; *p < 0.05.
